# Niche Differentiation of Comammox *Nitrospira* in the Mudflat and Reclaimed Agricultural Soils Along the North Branch of Yangtze River Estuary

**DOI:** 10.3389/fmicb.2020.618287

**Published:** 2021-01-14

**Authors:** Xinxin Wang, Lu Lu, Xue Zhou, Xiufeng Tang, Lu Kuang, Junhui Chen, Jun Shan, Huijie Lu, Hua Qin, Jonathan Adams, Baozhan Wang

**Affiliations:** ^1^College of Environmental Science and Engineering, China West Normal University, Nanchong, China; ^2^Department of Environmental Engineering, College of Resources and Environmental Sciences, Nanjing Agricultural University, Nanjing, China; ^3^State Key Laboratory of Soil and Sustainable Agriculture, Institute of Soil Science, Chinese Academy of Sciences, Nanjing, China; ^4^College of Agricultural Engineering, Hohai University, Nanjing, China; ^5^Key State Laboratory of Subtropical Silviculture, Zhejiang A&F University, Hangzhou, China; ^6^Key Laboratory of Environment Remediation and Ecological Health, Ministry of Education, College of Environmental Resource Sciences, Zhejiang University, Hangzhou, China; ^7^School of Geography and Ocean Science, Nanjing University, Nanjing, China; ^8^Key Lab of Microbiology for Agricultural Environment, Ministry of Agriculture, College of Life Sciences, Nanjing Agricultural University, Nanjing, China

**Keywords:** complete ammonia oxidizer, estuary, *amoA* gene, community structure, niche differentiation, soil property, co-occurrence network

## Abstract

The discovery of complete ammonia oxidation (comammox), oxidizing ammonia to nitrate via nitrite in a single organism, has redefined the traditional recognition of the two-step nitrification driven by two functional groups (ammonia-oxidizing and nitrite-oxidizing microorganisms). However, the understanding of the distribution and niche differentiation of comammox *Nitrospira* in the estuarine mudflats and their reclaimed agricultural soils is still limited. Here, we investigated the abundance, diversity and community structures of comammox *Nitrospira* in the mudflats and the reclaimed agricultural soils in the northern Yangtze River estuary. Quantitative PCR showed the abundances of *amoA* genes of comammox were lower than that of ammonia-oxidizing bacteria (AOB) in nearly all samples. Amplicon sequencing of *amoA* genes revealed that the community structures of comammox *Nitrospira* were significantly (*P* < 0.001) different between the original mudflats and the reclaimed agricultural soils, indicating niche differentiation among comammox *Nitrospira* clades (clade A.1, clade A.2, and clade B). The clade A.1 was the dominant group of comammox *Nitrospira* in the mudflats, while clade B predominated in the agricultural soils. However, the members of clade A.2 could be clearly divided into two groups, the mudflat-preferred and agricultural soil-preferred groups, suggesting more complicated ecological preferences within this sub-clade. Furthermore, it was demonstrated that salinity, organic matter (OM) and NO_3_^–^-N had a significantly influence on the distribution of comammox *Nitrospira* in the estuarine environment. Clade A.1 and nearly half members of clade A.2 were positively correlated with salinity, and negatively correlated with the concentrations of OM and NO_3_^–^-N. In contrast, the clade B and the other half members of clade A.2 showed the exact opposite pattern: a negative correlation with salinity and positive correlation with OM and NO_3_^–^-N. The co-occurrence network demonstrated that the operational taxonomic units (OTUs) within the same (sub-)clade were mostly positively correlated, indicating the similar niche preferences among the members from the same (sub-)clade of comammox *Nitrospira*. Taken together, our results revealed the niche differentiation of comammox *Nitrospira* in estuarine ecosystems where salinity and OM were the primary factors responsible for the distinct ecological distribution patterns.

## Introduction

Nitrification, the microbial conversion of ammonia to nitrate, is a central process in the global nitrogen cycle (Gruber and Galloway, 2008). For more than a century, nitrification was considered to be divided into two sequential step (Winogradsky, 1892), the ammonia oxidation (from ammonia to nitrite) performed by ammonia-oxidizing bacteria (AOB) and archaea (AOA) (Konneke et al., 2005), and the nitrite oxidation (from nitrite to nitrate) performed by nitrite-oxidizing bacteria (NOB). However, the recent discovery of complete ammonia oxidization (comammox) has broken this classical tenet (Daims et al., 2015; van Kessel et al., 2015). These novel comammox bacteria, affiliated to the genus *Nitrospira* (lineage II), contain all the necessary genes involved in the oxidation of ammonia and nitrite and thus can completely oxidize ammonia to nitrate in a single microorganism (Daims et al., 2015; van Kessel et al., 2015; Palomo et al., 2018). Phylogeny based on *amoA* gene encoding the alpha subunit of ammonia monooxygenase (AMO) revealed that comammox *Nitrospira* could be separated into clade A and clade B (Daims et al., 2015). Clade A could be further subdivided into two monophyletic groups (clade A.1 and A.2) (Xia et al., 2018). However, currently the ecological distribution of these phylotypes and their potential importance in the global nitrogen and carbon cycling remain poorly understood.

Metagenomic analysis and molecular surveys primarily based on amplicon sequencing of *amoA* genes demonstrated the widespread distribution of comammox *Nitrospira* in diverse habitats, including agricultural soils (Li et al., 2019; Zhao et al., 2019), groundwater-fed rapid sand filters (Palomo et al., 2016; Bartelme et al., 2017; Fowler et al., 2018), drinking water systems (Pinto et al., 2016; Wang et al., 2017), freshwater systems (Liu et al., 2020), wastewater treatment plants (WWTPs) (Pjevac et al., 2017; Annavajhala et al., 2018) and sediments (Yu et al., 2018), but not yet in marine ecosystems (Daims et al., 2015). The abundance of comammox *Nitrospira* was comparable to or even higher than those of AOA and AOB in some agricultural soils (Li et al., 2019) and engineered systems (Wang et al., 2018). Kinetic study indicates that the comammox species *N*. *inopinata* is more competitive in highly oligotrophic environments (Kits et al., 2017). Likewise, Xu Y. et al. (2020) recently found that eutrophication might inhibit the growth of comammox organisms in lake sediment. However, Wang J. et al. (2019) reported that the abundance of comammox bacteria was positively related to the amount of N fertilizer along a fertilization gradient in soil, and the two monophyletic clades (clade A and clade B) showed opposite responses to nutrient input. Recent studies also suggested the distinct distribution of clade A and clade B in different environments (Xia et al., 2018; Xu S. et al., 2020), which might be determined by multiple environmental factors (Hu and He, 2017; Shi et al., 2018; Roots et al., 2019; Xu Y. et al., 2020).

Estuary and coastal environments are the interface between land and sea serving as the hub of energy and material flows (Jickells, 1998). Over the past several decades, large-scale land reclamation of mudflats in estuaries has provided great economic benefits (Wang and Wall, 2010), but the nutrient input threatened the environment of estuarine ecosystems (Jickells, 1998; Chai et al., 2006). Nitrification coupling to denitrification or anaerobic ammonium oxidation is potentially an effective bioremediation pathway to remove excessive reactive nitrogen in these nitrogen-enriched environments (Rysgaard et al., 1996; Gruber and Galloway, 2008). Thus, the distribution and activity of ammonia-oxidizing microorganisms has become a subject of considerable interest (Wankel et al., 2011; Zheng et al., 2014). Previous studies have demonstrated that salinity, NH_4_^+^-N, organic carbon and other factors contribute to the niche differentiation of ammonia-oxidizing microorganisms (AOA and AOB) (Liu et al., 2013; Zhou et al., 2018). Recently, some studies suggested that comammox *Nitrospira* were quite abundant in the tidal flat sediments of estuaries (Sun et al., 2020), even as much so as AOA or AOB (Xia et al., 2018; Jiang et al., 2019). However, the distribution of comammox along a salinity gradient in estuarine continuum and the population dynamics in response to the land reclamation from mudflat to agricultural soils are still unknown.

The Yangtze River is the third-longest river of the world in terms of water volume, and carries a high biogeochemical flux of N nutrients (Zhu, 2005). In this study, quantitative PCR (qPCR) and Illumina HiSeq-based sequencing of *amoA* genes were employed to investigate the distribution and diversity of comammox *Nitrospira* in the original mudflat and the reclaimed agricultural soils along the north branch of Yangtze River estuary. Our aim is to reveal the niche differentiation of comammox *Nitrospira* phylotypes, and to explore potential factors driving the environmental distributions of comammox *Nitrospira* in estuarine ecosystems.

## Materials and Methods

### Soil Sampling and Physiochemical Analysis

The sampling sites were along the north branch of the Yangtze estuary, located in Jiangsu Province, China (Supplementary Figure S1). This region is characterized by a typical subtropical wet monsoon climate. The mean annual temperature in this region is 15.1°C and the mean annual rainfall is 1,040 mm. Since the 1990s, a large part of upper intertidal mudflats was reclaimed to agricultural fields with a rapeseed-rice rotation system. We sampled 22 locations including both mudflat sites (M1-M11) and the agricultural sites (A1-A11) in April 2017 (Supplementary Figure S1). Each mudflat site was ≤ 200 m apart from the paralleled agricultural site and located in the supratidal zone, which remain non-flooded condition at most time. At each sampling site, three replicates were collected, and each replicate contained five soil cores (0–5 cm). The soil samples were freeze-dried and passed through a 2 mm sieve and stored at −20°C for following DNA extraction and biochemical analysis.

Soil pH and salinity were measured using a soil-water suspension (1:2.5 soil/water) after shaking for 30 min using a Mettler-Toledo pH/EC meter (SevenMulti S40, Switzerland) (Wang et al., 2014). Organic matter (OM) in soils was determined using the K_2_Cr_2_O_7_ oxidation-reduction titration method. Nitrate (NO_3_^–^-N) and ammonium (NH_4_^+^-N) were extracted with 2 M KCl at a soil-to-solution ratio of 1:5 and measured with a continuous flow analyzer (Skalar Inc., Breda, Netherlands).

### DNA Extraction and Quantitative PCR

Total soil DNA was extracted from each sample using the Fast DNA spin kit for soil (MP Biomedicals, Santa Ana, CA). DNA concentration was then quantified by a Nanodrop ND-1000 UV-vis Spectrophotometer (Thermo Scientific, Wilmington, DE, United States). The absolute abundances of comammox *Nitrospira*, AOA and AOB in each soil sample were determined by using a CFX96 Optical Real-Time Detection System (Bio-Rad Laboratories, Inc., Hercules, CA, United States). The primer pairs of Ntsp-*amoA* 162F (5′-GGATTTCTGGNTSGATTGGA-3′)/Ntsp-*amoA* 359R (5′-WA GTTNGACCACCASTACCA-3′) (Fowler et al., 2018), Arch-*amoA*F (5′-STAATGGTCTGGCTTAGACG-3′)/Arch-*amoA*R (5′-GCGGCCATCCATCTGTATGT-3′) (Francis et al., 2005), and *amoA*1F (5′-GGGGTTTCTACTGGTGGT-3′)/*amoA*2R (5′-CCCCTCKGSAAAGCCTTCTTC-3′) (Rotthauwe et al., 1997) were used for the qPCR of comammox, AOA and AOB *amoA* genes, respectively. Each sample was amplified in triplicate 20 μl containing 10 μl of 2 × SYBR Premix Ex Taq^TM^ (Takara, Dalian, China), 0.5 μl of each primer (10 μM) and 10 ng of template DNA. The qPCR reactions were performed using the following thermocycling protocol: 95°C for 3 min, followed by 40 cycles of 95°C for 10 s, 48°C (55°C for AOA/AOB *amoA*) for 30 s and extension at 72°C for 45 s, and melt curve (65–95°C) 0.2°C/s gradient. The qPCR standards were generated using serial dilutions (from 10^1^ to 10^9^ copies) of plasmid DNA from one representative clone containing *amoA* genes of comammox *Nitrospira*, AOA or AOB. The amplification efficiencies were determined as above 96% with *R*^2^ values of 0.995–0.999. Additionally, melting curve analyses and agarose gel electrophoresis were conducted to determine the specificity of the amplified products.

### Amplicon Sequencing and Bioinformatic Analysis

For amplicon sequencing, the *amoA* gene of comammox *Nitrospira* was amplified using primers Ntsp-*amoA* 162F/Ntsp-*amoA* 359R, and the forward primers were modified to contain a unique barcode (10 bp) at the 5′ end for each sample. PCR reactions were performed in a 50 μl mixture containing 25 μl of 2 × Rapid Taq Master Mix (Vazyme, Nanjing, China), 2 μl of each primer (10 μM) and 20 ng of template DNA. The amplification conditions were as follows: 95°C for 3 min, and 30 cycles of 95°C for 15 s, 48°C for 30 s, and 72°C for 30 s, followed by 72°C for 5 min. Each sample was amplified in triplicate, and the PCR products were purified using AxyPrep gel extraction kit (AxyGen, Hangzhou, China) and quantified by Nano-Drop ND-1000 spectrophotometer. Purified amplicons were pooled together in equimolar ratios into one single tube and sent for paired-end sequencing (2 × 150 bp) on an Illumina HiSeq platform (Illumina, San Diego, United States) at Majorbio Bio-Pharm Technology Co., Ltd., Shanghai, China.

Raw data were processed using the Quantitative Insight into Microbial Ecology (QIIME) toolkit^1^ (Caporaso et al., 2010). Paired-end reads were first merged using the FLASH tool based on the matched overlapping regions (Magoc and Salzberg, 2011). Sequences were quality filtered (quality score > 20), then were split into libraries of samples by specific barcode sequences. Chimera detection and elimination was accomplished using uchime *de novo* function, which was based on self-sequences (Edgar et al., 2011). The remaining sequences sharing ≥ 95% identity were clustered into one operational taxonomic unit (OTU). The representative sequence of each OTU was blasted against NCBI-nr database, and only the candidates with the hits of *amoA* or *pmoA* genes (comammox *amoA* gene might be wrongly annotated as *pmoA* gene before the discovery of comammox in 2015; Daims et al., 2015; van Kessel et al., 2015) were collected and further confirmed by using phylogenetic analysis with the reference comammox *amoA* sequences (Lin et al., 2020). QIIME’s scripts alpha_rarefaction. py and beta_diversity_through_plots. py were used to compute alpha and beta diversity values, and all samples were uniformed to a certain number of sequences per sample. Phylogenetic tree was constructed using Maximum Likelihood method and calculated in MEGA 6.0 with 100 bootstrap replicates (Tamura et al., 2013).

### Co-occurrence Network Analysis

To reduce rare OTUs in the data set, only the OTUs with relative abundance ≥ 0.01% and occurring in more than 50% of all samples were kept for co-occurrence network analysis (Ma et al., 2016; Delgado-Baquerizo et al., 2018). In order to reduce the spurious correlation associated with compositional bias, we calculated all possible correlation coefficients using SparCC method (Kurtz et al., 2015). We considered statistical significance with cutoff at *P* < 0.01 (adjusted with Benjamini-Hochberg false discovery rate (FDR) control procedure) (Yoav Benjamini et al., 2006) and correlations with cutoff at *r* ≥ 0.6 (Junker and Schreiber, 2008; Barberan et al., 2012). Network topological parameters were calculated with “igraph”^2^ and “WGCNA” package^3^. Network images were generated with Gephi 0.9.1^4^ with the Fruchterman-Reingold layout. The nodes with the highest degree centrality (degree > 10) in were defined as hub nodes, and the other nodes were defined as peripheral nodes (Ma et al., 2018).

### Statistical Analysis

ANOVA analysis was performed to evaluate differences in soil physiochemical properties, gene copies, diversity index among samples using SPSS Statistics 19.0 (IBM Corporation, Armonk, NY, United States). Spearman’s correlations were used to reveal significant correlations between the diversity, the relative abundance of each OTUs and soil physiochemical properties. Non-metric multidimensional scaling (NMDS) and pairwise comparisons (ADONIS, ANOSIM, and MRPP) were conducted to display differences in comammox *Nitrospira* community composition based on the Bray-Curtis dissimilarity matrix of detected OTUs using the “vegan” package in R3.5.4. Response Ratio (RR) (Luo et al., 2006) was performed to explain the changes in the relative abundance of OTU between niches. Mantel test (“vegan” package in R 3.5.4), Multiple Regression Tree analysis (MRT) (“mvpart” package in R 2.1.5) and Constrained Correspondence Analysis (CCA) (Canoco 5 v. 5.02) were conducted to analysis the relationships between environmental properties and community structure. Distance-based multivariate analysis (DistLM) (Mcardle and Anderson, 2001) was also performed to determine the effect of soil properties on community structure of comammox *Nitrospira*, the sequential and marginal tests were analyzed to determine the statistical significance and the relative contribution of each environmental variable.

### NCBI Sequence Accession Numbers

Amplicon date was deposited in the Sequence Read Archive (SRA) at NCBI under the accession number PRJNA644509. The OTUs representative *amoA* genes were submitted to the NCBI GenBank under accession no. MT395049 to MT395142.

## Results

### Soil Physiochemical Properties

**FIGURE 1:**
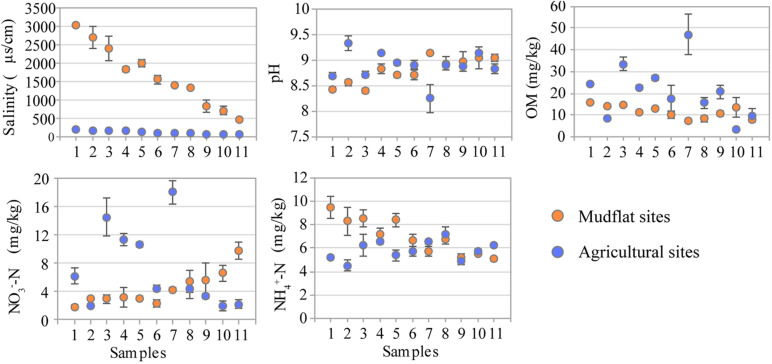
Soil physiochemical properties of mudflat and agricultural soils.

Soil salinity in the mudflats was typically high, showing a salinity gradient descending from the lower estuary (M1, 3040.0 μS/cm) to the upper estuary (M11, 391.0 μS/cm) (*P* < 0.01), which remarkably decreased (*P* < 0.01) in reclaimed agricultural soil (46.2–217.0 μS/cm) (Figure 1 and Supplementary Table S1). The soil pH was alkaline in all samples, ranging from 8.44 to 9.14 in mudflat and 7.88 to 9.59 in agricultural soils. The organic matter (OM) varied from 7.93 mg/kg to 15.93 mg/kg in mudflat sites with the mean value 11.65 mg/kg, which significantly increased in agricultural sites (range 8.26–46.97 mg/kg, mean 20.92 mg/kg). Similarly, the average content of NO_3_^–^-N in agricultural soils was also higher than that of mudflat (7.18 vs. 4.38 mg/kg). There was no significant difference in the concentration of NH_4_^+^-N between the mudflat (5.08–9.50 mg/kg) and agricultural sites (4.52–7.17 mg/kg) (Figure 1 and Supplementary Table S1).

### Abundance of the *amoA* Genes of Ammonia Oxidizers

**FIGURE 2:**
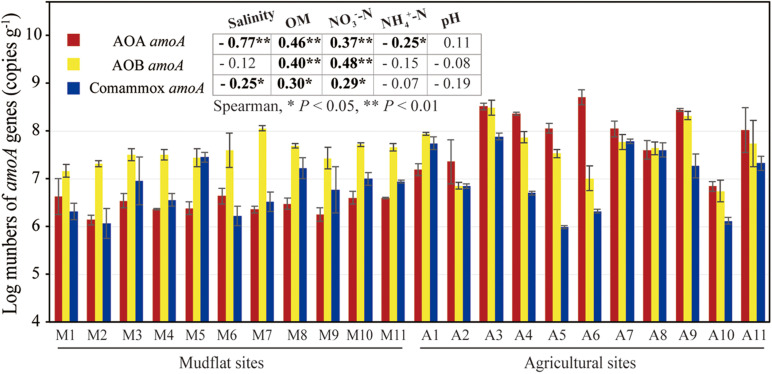
AOA, AOB and comammox *Nitrospira amoA* gene copy numbers in mudflat (M1–M11) and reclaimed agricultural soils (A1–A11). The mean values of *amoA* copy numbers were obtained from triplicate samples. Embedded chart shows the correlation between the *amoA* gene numbers and soil properties based on spearman rank (*P < 0.05, **P < 0.01).

The *amoA* gene abundance of comammox *Nitrospira*, AOA and AOB in each sample were quantified using qPCR assays (Figure 2). In mudflat, the number of comammox *Nitrospira amoA* genes ranged from 1.93 × 10^6^ to 3.00 × 10^7^ copies g^–1^ soil, which were higher than or comparable to AOA *amoA* genes (1.45 × 10^6^ copies − 9.05 × 10^6^ copies g^–1^ soil) (Figure 2). The AOB *amoA* genes abundances were higher than counterparts of AOA and comammox *Nitrospira*, ranging from 1.58 × 10^7^ to 1.15 × 10^8^ copies g^–1^ soil, with the ratio of AOB/AOA *amoA* and AOB/comammox *Nitrospira amoA* varying from 1.75 to 45.59 and 1.08 to 34.73, respectively. In the agricultural soils, the comammox *Nitrospira amoA* gene abundances ranged from 9.76 × 10^5^ to 7.74 × 10^7^ copies g^–1^ soil (Figure 2). The number of AOA *amoA* genes reached to 7.38 × 10^6^ − 5.74 × 10^8^ copies g^–1^ soil. The AOB *amoA* genes ranged from 7.25 × 10^6^ to 3.48 × 10^8^ copies g^–1^ soil (Figure 2), and the ratio of AOB/AOA *amoA* and AOB/comammox *Nitrospira amoA* ranged from 0.03 to 1.05 (except site A1 was 5.22) and 1.02 to 36.09, respectively. Spearman correlation displayed that the *amoA* abundance of AOA and comammox *Nitrospira* were negatively correlated with salinity, and AOA *amoA* gene abundance also showed significant negative correlation with the concentration of NH_4_^+^-N (Figure 2). Additionally, the *amoA* gene abundances of all three groups of ammonia-oxidizing microorganisms exhibited positive relationship with OM and NO_3_^–^-N (Figure 2).

### Diversity and Community Structure of Comammox *Nitrospira*

**FIGURE 3:**
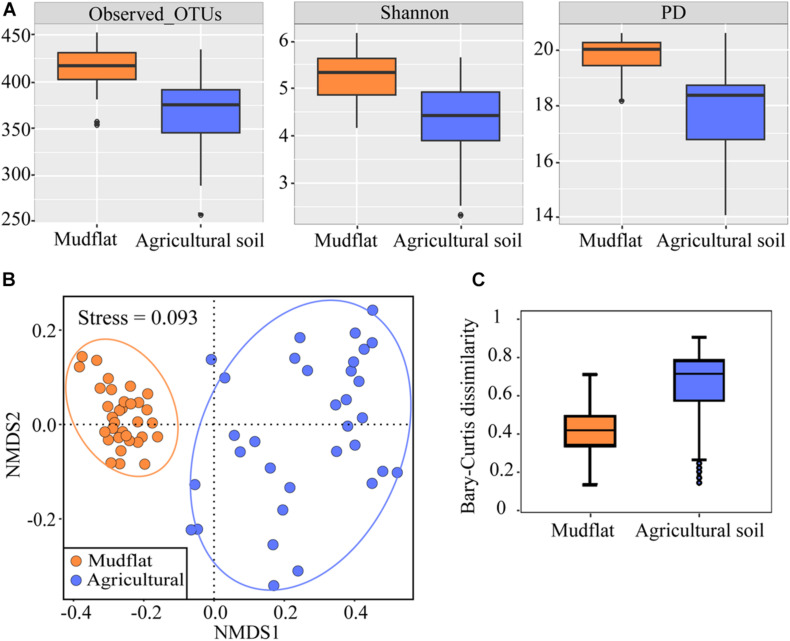
α-diversity and β-diversity of comammox *Nitrospira amoA* genes in mudflat and agricultural sites. **(A)** Number of observed OTUs, the Shannon index, and the PD values using OTU similarity thresholds of 95% for the *amoA* gene. **(B)** NMDS analysis based on Bray-Curtis dissimilarities of comammox *Nitrospira* communities. **(C)** Bray-Curtis dissimilarities between mudflat and agricultural soils, *P* < 0.01.

After filtering, a total of 7,307,681 high-quality comammox *Nitrospira amoA* gene sequences was obtained from all the 66 soil samples, which were then clustered into 546 OTUs at 95% sequence identity. Rarefaction curve of each soil sample gradually reached a plateau after 5,000 sequences, indicating that the dataset was sufficient for the diversity analysis of comammox *Nitrospira* in the samples studied (Supplementary Figure S2). The sequences in each sample were rarified to 20,006 for subsequent analysis. The α diversity of comammox *Nitrospira* in the mudflat was higher than that in the agricultural soils, both in phylotype richness (Observed OTUs and Shannon) and phylogenetic diversity (PD) (*P* < 0.01) (Figure 3A). NMDS demonstrated that soil comammox *Nitrospira* communities varied between habitats (Figure 3B), which was further supported by non-parametric multivariate analysis (ADONIS, *R*^2^ = 0.3997, *P* < 0.001; ANOSIM, *R*^2^ = 0.3997, *P* < 0.001; MRPP, *R*^2^ = 0.2019, *P* < 0.001) (Supplementary Table S2). The within-habitat pairs of Bray-Curtis dissimilarity revealed that the community dissimilarity was greater in agricultural than that in mudflat (Figure 3C).

**FIGURE 4:**
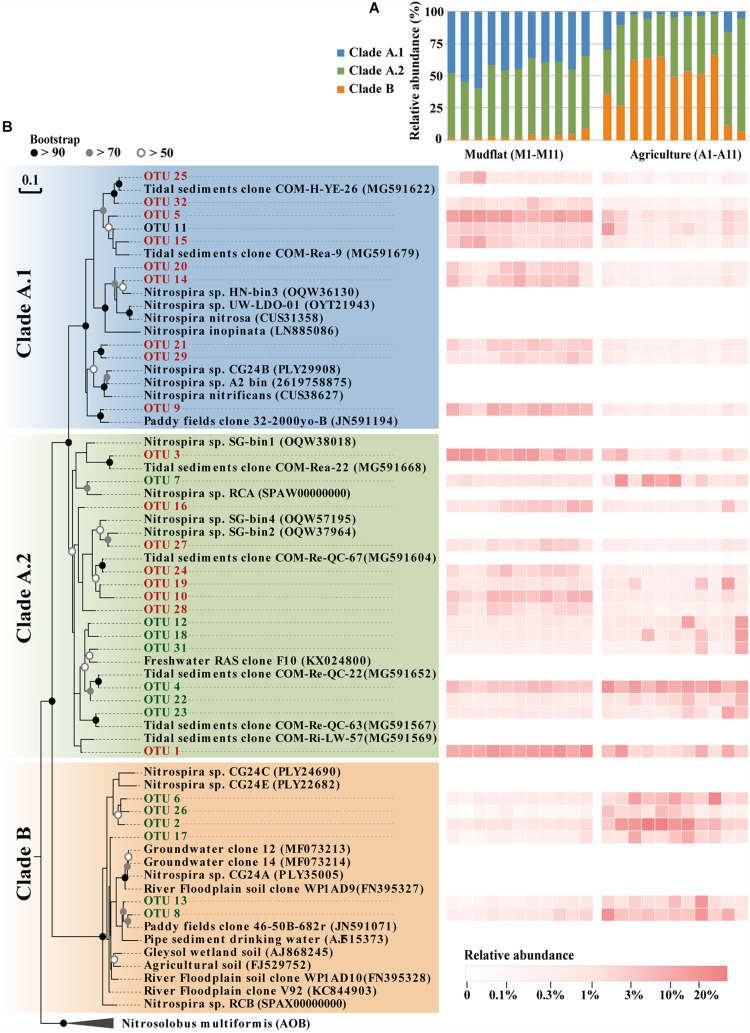
Community composition of comammox *Nitrospira* in different sites of this study. **(A)** Relative proportions of the different clades of comammox *Nitrospira* in 22 different sites samples. **(B)** Phylogenetic tree and relative abundance of comammox *Nitrospira amoA* genes sequences. Dominate OTUs with a relative abundance > 1% were included in the phylogenetic analysis, accounting for 74% of the total comammox *Nitrospira amoA* gene sequences. The log value of relative abundance (%) of individual taxa in each site was displayed as a heat map representation. The phylogenetic position of each representative OTU was shown in the phylogenetic tree on the left-hand side. OTUs labeled in red represent mudflat-preferred OTUs (MPOs), OTUs labeled in green represent agricultural soil-preferred OTUs (APOs).

Phylogenetic analysis demonstrated that 90.8–99.2% of comammox *Nitrospira amoA* gene sequences in the mudflat fell into clade A, and 34.1–59.6% of the sequences belonged to clade A.1 and 38.9–59.9% belonged to clade A.2 (Figure 4A). Clade B only accounted for 0.8–9.2% of comammox *Nitrospira* community in the mudflat soils (Figure 4A). However, the relative abundance of clade A.1 sharply decreased in the reclaimed agricultural soils compared to the mudflat soils, and clade B was the most dominant lineage of comammox *Nitrospira* in most agricultural soils with the exception of sites A2, A10, and A11 (Figure 4A). Additionally, the relative abundance of clade A.2 was much higher than that of clade A.1 in the agricultural soils (Figure 4A). However, it did not mean that the relative abundance of each OTU from clade A.2 in the agricultural soils was higher than that in the mudflat. For instance, OTU1, 3, 10, 16, 24, 27, and 28 were primarily distributed in the mudflat with the relative abundance of 1.1–14.0% that were significantly higher (*P* < 0.05) than those in the agricultural soils, whereas OTU4, 7, 12, 18, 19, 22, 23, and 31 were much more abundant (*P* < 0.05) in the agricultural soils than in the mudflat soils (Figure 4B and Supplementary Table S3).

### Correlation Between Soil Properties and Diversity and Community Structure of Comammox *Nitrospira*

Spearman analysis demonstrated that the α-diversity (Observed OTUs, Shannon index and Phylogenetic diversity) of comammox *Nitrospira* communities was significantly positively correlated with salinity (*P* < 0.05), but was negatively correlated with content of OM and NO_3_^–^-N (*P* < 0.05) in the estuarine environments (Table 1). Mantel test and multivariate regression tree (MRT) analysis also suggested the significant influence of soil variables on comammox *Nitrospira* community structure in the estuary studied with the salinity at the top of the list, followed by OM and NO_3_^–^-N (Table 1 and Supplementary Figure S3). DistLM analysis further revealed that salinity, OM and NO_3_^–^-N could explain 27.43, 8.92, and 2.66% of the community variation of comammox in the estuarine environments (Supplementary Table S4).

**TABLE 1:** Relationship between soil physiochemical properties and abundance and community diversity of comammox *Nitrospira*.

	**α-diversity of comammox^*a*^**			**β-diversity of comammox^*b*^**	
	**Observed_ species**	**Shannon**	**PD**	**Bray_ Curtis**	**Weighted_ unifrac**
Salinity	0.49**	0.4**	0.55**	0.32**	0.30**
OM	−0.57**	−0.5**	−0.55**	0.36**	0.33**
NO_3_^–^-N	−0.37**	−0.33**	−0.38**	0.23**	0.27**
NH_4_^+^-N	0.07	0.19	0.16	–0.03	–0.01
pH	0.06	–0.04	–0.02	0.06	–0.02

^*a*^Spearman rank of α-diversity of comammox Nitrospira and soil properties. ^*b*^Mantel test between Bray_Curtis distance as well as weighted_unifrac of comammox Nitrospira community and soil properties. **P < 0.01.

**FIGURE 5:**
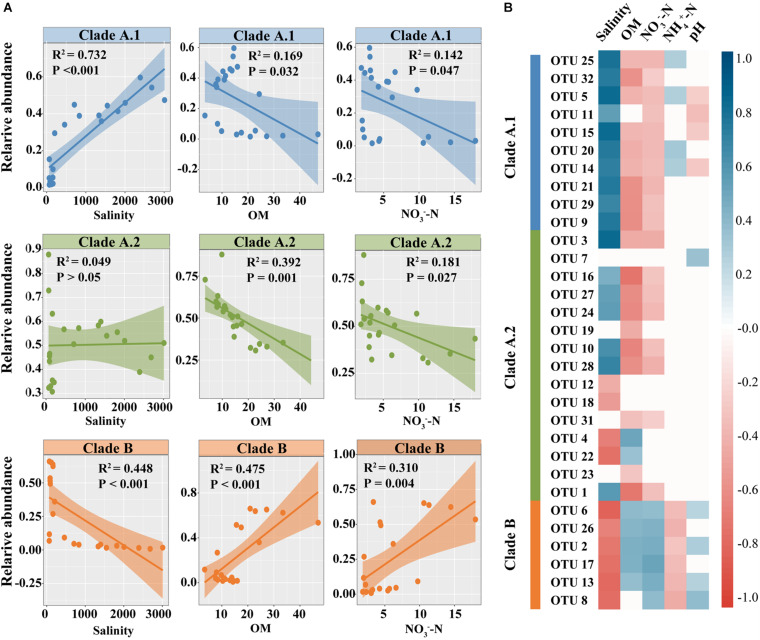
Relationships between relative abundance of comammox *Nitrospira* and soil properties. **(A)** Linear regression analysis between relative abundance of different comammox clades and environmental properties. **(B)** Spearman correlations of dominant OTUs (abundance > 1%) with soil properties in all samples, displayed as heatmaps. Color bar indicates correlation coefficients. Only significant correlations (*P* < 0.05) were shown.

Linear regression analysis revealed that relative abundance of clade B showed significantly negative relationship with salinity (*R*^2^ = 0.448, *P* < 0.01), but positive relationship with OM (*R*^2^ = 0.475, *P* < 0.01) and NO_3_^–^-N (*R*^2^ = 0.310, *P* < 0.01) (Figure 5A). On the contrary, clade A.1 was positively correlated with salinity (*R*^2^ = 0.732, *P* < 0.01), but negatively correlated with OM (*R*^2^ = 0.169, *P* < 0.05) and NO_3_^–^-N (*R*^2^ = 0.142, *P* < 0.05). However, clade A.2 showed significant negative correlation with OM (*R*^2^ = 0.392, *P* < 0.01) and NO_3_^–^-N (*R*^2^ = 0.270, *P* < 0.01), but had no significant correlation with salinity on the whole sub-clade level (*P* > 0.05) (Figure 5A). Nonetheless, although there was no significant relationship between the distribution of clade A.2 and salinity on the whole sub-clade level, the OTUs of clade A.2 could be roughly divided into two groups according to their positive or negative relationship with salinity, respectively (Figure 5B). Taking the dominant OTUs for example, OTU 1, 3, 10, 16, 24, 27, and 28 were significantly positively related to salinity (*P* < 0.05), while OTU 4, 12, 18, and 22 showed negative relationship with salinity (*P* < 0.05) (Figure 5B). The similar patterns were also observed for most of the rare OTUs (0.1% < relative abundance <1%) within the clade A.2 (Supplementary Figure S4). All these results were also supported by the canonical correspondence analysis (CCA) (Supplementary Figure S5).

### Co-occurrence Network of Comammox *Nitrospira* Species

**FIGURE 6:**
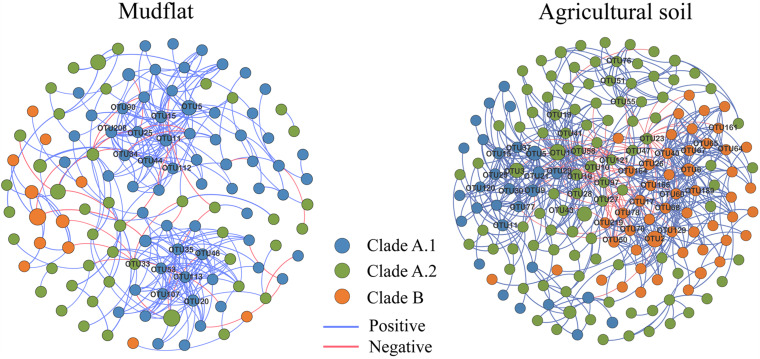
Co-occurrence network of comammox *Nitrospira* species. The nodes represent OTUs, the relationship of two OTUs in the network was shown by an edge (Coefficient *r* ≥ 0.6, significant *P* ≤ 0.01). Nodes in different colors represent different clades of comammox *Nitrospira*, and the size of the nodes was proportional to their relative abundance; the pink and blue edges represent the positive and negative correlations, respectively, and the thickness of each connection between two nodes is proportional to the value of correlation coefficient. Hub nodes of co-occurrence were labeled in networks.

According to the statistically significant difference on the relative abundance of OTUs between the mudflat and agricultural soils (Response ratio, *P* < 0.05), we grouped the OTUs of comammox *Nitrospira* into three ecological clusters, mudflat-preferred OTUs (MPOs), agricultural soil-preferred OTUs (APOs), and a small part of ubiquitous OTUs (UBOs) (Supplementary Table S3). To identify the co-occurrence patterns of comammox *Nitrospira* species, we constructed a network based on significant (*P* < 0.01) correlations (r ≥ 0.6) among species pairs in mudflat and agricultural soils, respectively (Figure 6). The two empirical networks were verified as non-random and scale-free, based on topological comparison with random network and a power-law distribution pattern (*P* < 0.001) of degree (Supplementary Table S5 and Supplementary Figure S6).

The connection among the members in the network of agricultural soils seemed to be much tighter than that of mudflat soils, since the former one contained more nodes (i.e., OTUs), more links (i.e., correlation between two OTUs), higher average degree, and less average path length that those of the latter one (Figure 6 and Supplementary Table S5). In the network of mudflat, the members of clade A.1 had the highest average degree (6.39) compared to clade A.2 (2.95) and clade B (2.67), and most (15 in 16) of hub nodes (degree > 10) belonged to clade A.1 (Supplementary Table S6). However, in the network of agricultural soils, the average degree of clade B (8.98) were higher than those of clade A.1 (7.38) and clade A.2 (5.48), and a total of 47 nodes were determined as hub species, including 19 OTUs of clade B, 17 OTUs of clade A.2 and 11 OTUs of clade A.1 (Supplementary Table S6).

The connections among the members in both networks were mostly positive (90.4% for the mudflat network and 89.5% for the agricultural soil network) (Supplementary Table S7 and Figure 6). It was noteworthy that the connections mostly occurred among the members within each clade (Mudflat: 84.9%; Agricultural soil: 83.1%) rather than those between each clade (Mudflat: 15.1%; Agricultural soil: 16.9%) (Supplementary Table S7). Furthermore, 92 and 100% of connections among the members within MPO cluster in the two networks were positive, and 100 and 93% within APO cluster were positive, but more than 70% of connections among the members between MPO and APO cluster were negative (Supplementary Table S8).

## Discussion

In this study, by using qPCR and high-throughput sequencing of comammox *Nitrospira amoA* genes, we determined the distribution of comammox *Nitrospira* in the mudflat and the reclaimed agricultural soils along the north branch of Yangtze river estuary, a typical land-sea transitional area. The abundance, diversity and community composition of comammox *Nitrospira* showed significant differences between the mudflat and agricultural soils, which was influenced by the various soil properties.

Previous studies demonstrated the ubiquitous distribution of comammox *Nitrospira* in terrestrial ecosystems (Daims et al., 2015; van Kessel et al., 2015; Lawson and Lücker, 2017), but not yet in marine environment. Here, we detected abundant comammox *Nitrospira amoA* genes in the mudflats and the reclaimed agricultural soils, around 10^6^ to 10^7^ copies g^–1^ soil, which was however generally lower than those of canonical ammonia oxidizers, AOB and AOA. Not surprisingly, similar results also detected in previous studies (Yu et al., 2018; Sun et al., 2020). Considering the increased salinity gradient from estuarine area to coastal water and then to the open sea coupled with the distribution patterns of comammox *Nitrospira*, high salinity may be one of major factors inhibiting occurrence of comammox *Nitrospira* in the open sea (see below).

The anthropogenic land transformation dramatically affected the community assembly of comammox *Nitrospira* in the estuarine environments. In this study, clade A was revealed as the predominant lineage of comammox *Nitrospira* in the original mudflats and was mostly characterized as MPOs cluster, while clade B and some members of clade A.2 combinedly dominated the comammox *Nitrospira* community in the reclaimed agricultural soils and termed as APOs. This result was consistent with the previous studies that estuarine sediment and coastal waters preferred the clade A comammox *Nitrospira* and most of them belonged to subclade A.1, but clade B was negligibly detected in these environments (Yu et al., 2018; Jiang et al., 2019). On the contrary, considerable abundant *amoA* genes of clade A.2 (Xu S. et al., 2020) and clade B (Wang Z.H. et al., 2019) were enriched in agricultural soils, and the ^13^CO_2_-DNA-stable isotope probing proved the activity of clade A.2 (Li et al., 2019) and clade B (Wang Z.H. et al., 2019) in the nitrification of agricultural soils. These results suggested high diversity and strong niche differentiation of comammox in diverse environments.

Salinity and OM were revealed as the major factors determining the distribution of comammox in the estuarine environments in our study. The abundance of comammox *Nitrospira* were significantly negatively correlated with salinity (Figure 2), indicating high salinity could depress the activity of most comammox *Nitrospira* members, especially of clade B. This may to some extent explain why the niche of comammox *Nitrospira* could expand to estuarine environment (Yu et al., 2018) and coastal water (Xia et al., 2018), but not yet to the open sea, in which salinity is as high as around 35‰ and much higher than those of estuarine and coastal waters. Moreover, we presumed that OM was also a key factor driving the distribution of APO members (clade B and a part of clade A.2), besides the salinity restriction. For instance, particularly, in two newly reclaimed (3 years) agricultural sites A10 and A11 with similar salinity with but lower OM than the rest of agricultural soils, the relative abundance of clade B were as few as that of the mudflat with low OM but high salinity (Figure 4A). Thus, OM might be the principal factor in governing niche adaptation of clade B in agricultural soils. Although currently, there is no direct evidence for the growth of clade B on organic carbon, the genomic analysis revealed the presence of enzymes and pathways involved in different carbon source degradation, suggesting potential mixotrophic lifestyle for clade B comammox *Nitrospira* as reported for other *Nitrospira* spp. (Watson et al., 1986; Daims et al., 2001; Spieck et al., 2006). Additionally, pH and NH_4_^+^-N had previously also been recognized as main factors impacting the diversity and activity of ammonia oxidizers in terrestrial environments (Prosser and Nicol, 2012; Shi et al., 2020; Xu Y. et al., 2020). However, we did not detect significant effect of pH and NH_4_^+^-N on the abundance and community of comammox *Nitrospira* in our study, that might due to the not wide range of pH (7.88–9.59) and NH_4_^+^-N (4.5–9.5 mg/kg) among our all samples (Supplementary Table S1). All these conclusions warrant the future physiological and biochemical study of pure cultures of comammox *Nitrospira*. However, it should be pointed out that we did not determine more comprehensive soil properties, such as distribution of particle size and concentration of metal ions, which could also influence the distribution of comammox *Nitrospira*.

Co-occurrence network analysis is a powerful tool in exploring the correlation or interaction among the members of microbial communities (Weiss et al., 2016), which has been performed to explore community patterns of bacteria and fungi (Mendes et al., 2014; Ma et al., 2016), as well as ammonia oxidizers (Shi et al., 2020; Sun et al., 2020). More nodes, more links and higher average degree as well as shorter average path length were found in the agricultural soil network, indicating a more complex co-occurrence pattern in agricultural soil. But a more complex co-occurrence pattern did not necessarily equal more ecological interactions among the members of comammox *Nitrospira* in the agricultural soils than the mudflat. In our study, positive correlations among comammox *Nitrospira* species predominated in the networks of both the mudflat and agricultural soils. As previously reported positive connection might be due to cross-feeding, co-aggregation, co-colonization, niche overlap or other reasons (Faust and Raes, 2012). It was hard to distinguish between true ecological interactions and other non-random processes (for example, cross-feeding vs. niche overlap) from most co-occurrence networks, but similar niche preference could be the reason why closely related species co-occur more often than those distantly related species (Chaffron et al., 2010; Faust and Raes, 2012). It was noteworthy that all comammox members depend on the energy generated from complete oxidation of ammonia to nitrate, and comammox *Nitrospira* within the same subclade were more connected. Therefore, we presumed that positive correlations among comammox *Nitrospira* of the MPOs or APOs groups largely due to the shared niche preference (Supplementary Table S8), but not biotic interaction as observed in macro-ecological co-occurrence networks (Freilich et al., 2018).

Taken together, this study revealed the ubiquitous distribution and significant niche differentiation among different (sub-)clade of comammox, clade A.1, clade A.2, and clade B, in the estuarine environment and reclaimed agricultural soils. Moreover, salinity and the concentration of OM were revealed as the major factors driving the distribution of each (sub-)clade in the estuarine environments, but more physiological and biochemical studies of pure cultures are needed to prove these conclusions.

## Data Availability Statement

The datasets presented in this study can be found in online repositories. The names of the repository/repositories and accession number(s) can be found in the article/Supplementary Material.

## Author Contributions

BW proposed the idea. XW and BW designed the main idea of this work. XZ provided the resources of this study. XW and XT performed the experiments. XW, LL, and LK contributed to the interpretation of data for the work. XW drafted the manuscript. BW, LL, JA, JS, HL, HQ, and JC revised it critically for important intellectual content. All authors contributed to the article and approved the submitted version.

## Conflict of Interest

The authors declare that the research was conducted in the absence of any commercial or financial relationships that could be construed as a potential conflict of interest.

## Footnotes

1 http://qiime.org/

2 https://igraph.org/

3 https://horvath.genetics.ucla.edu/html/CoexpressionNetwork/Rpackages/WGCNA/

4 http://gephi.github.io/
