# Supplementary material for: Niche Differentiation of Comammox Nitrospira in the Mudflat and Reclaimed Agricultural Soils Along the North Branch of Yangtze River Estuary
Source: Front Microbiol. 2021 Jan 14;11:618287. doi: 10.3389/fmicb.2020.618287 (PMC7873905; doi:10.3389/fmicb.2020.618287)
Supplement: Supplementary file 1 [file Data_Sheet_1.docx]

***Supplementary Figures***


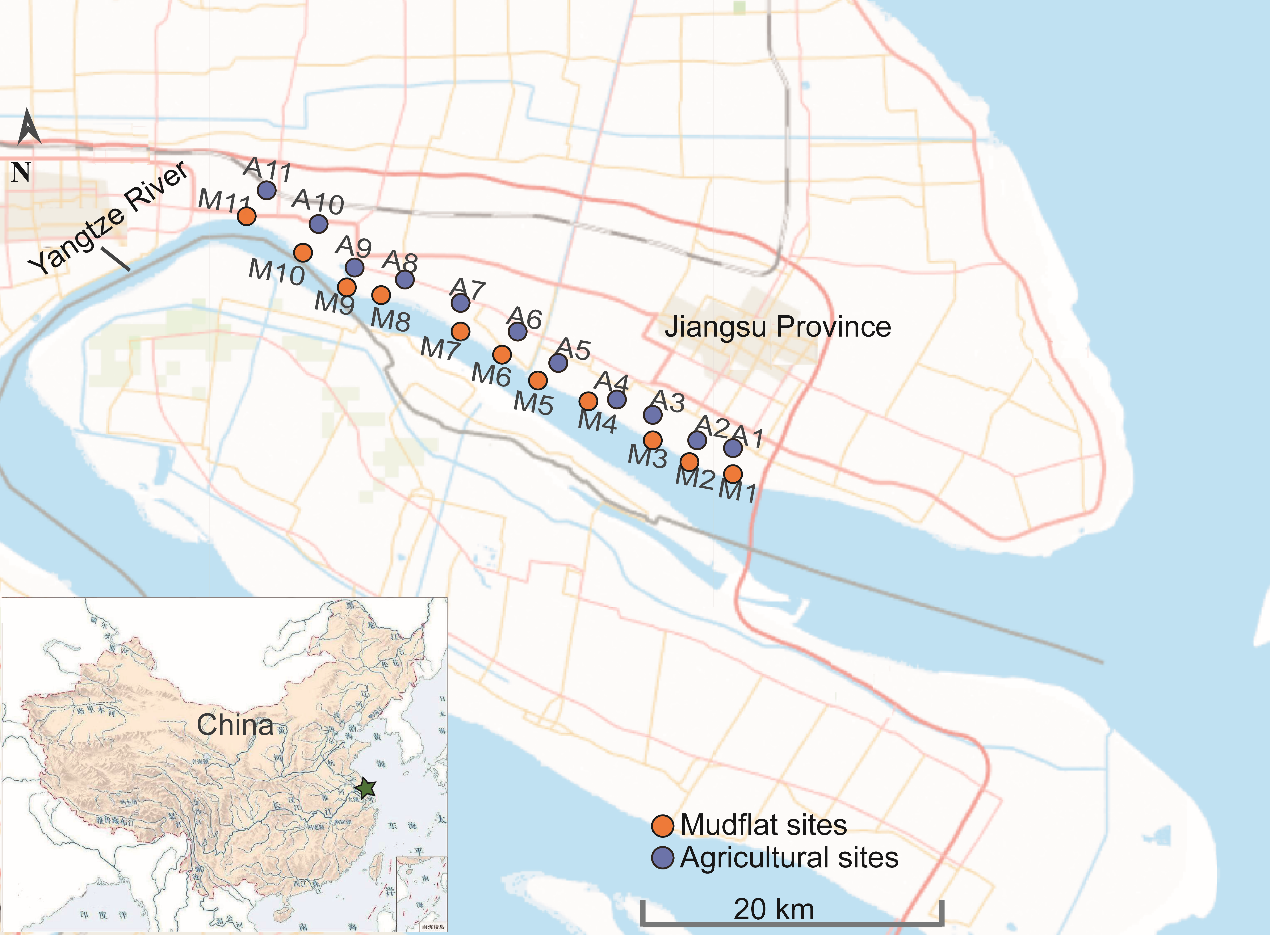


**Supplementary Figure S1 Sampling locations along the Yangtze River estuary.** Sampling sites marked

with orange circle at mudflat and blue circle at the reclaimed agricultural soils.


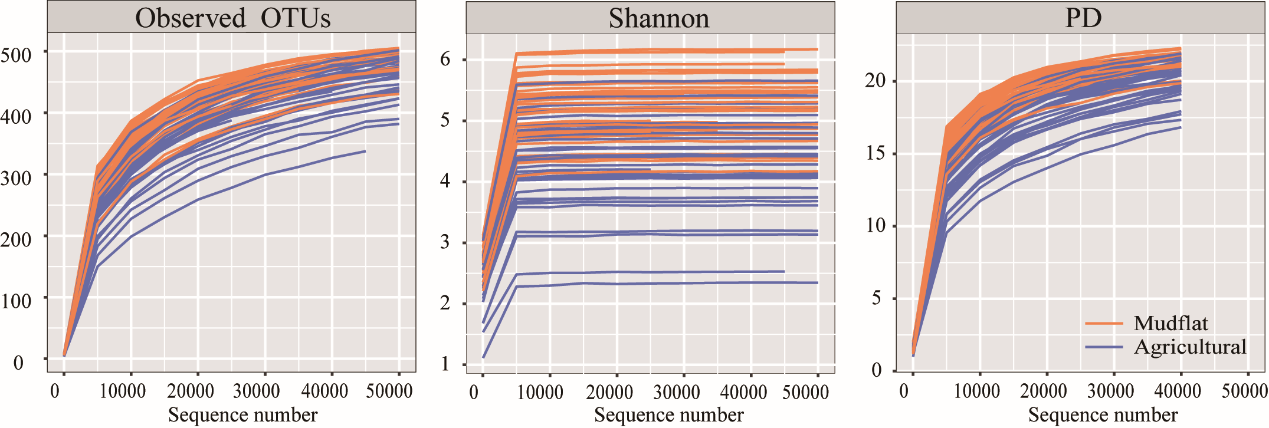


**Supplementary Figure S2 Rarefaction curves of the observed species.** The Shannon index, and the PD values using OTU similarity thresholds of 95% for the *amoA* gene of comammox *Nitrospira*.

**Supplementary**
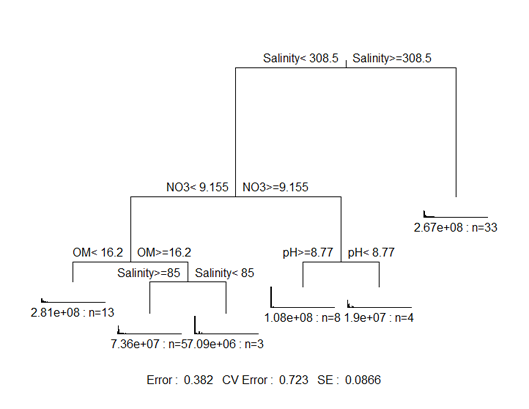
**Figure S3 MRT analysis of the relationship between soil properties and community structure of comammox *Nitrospira*.**


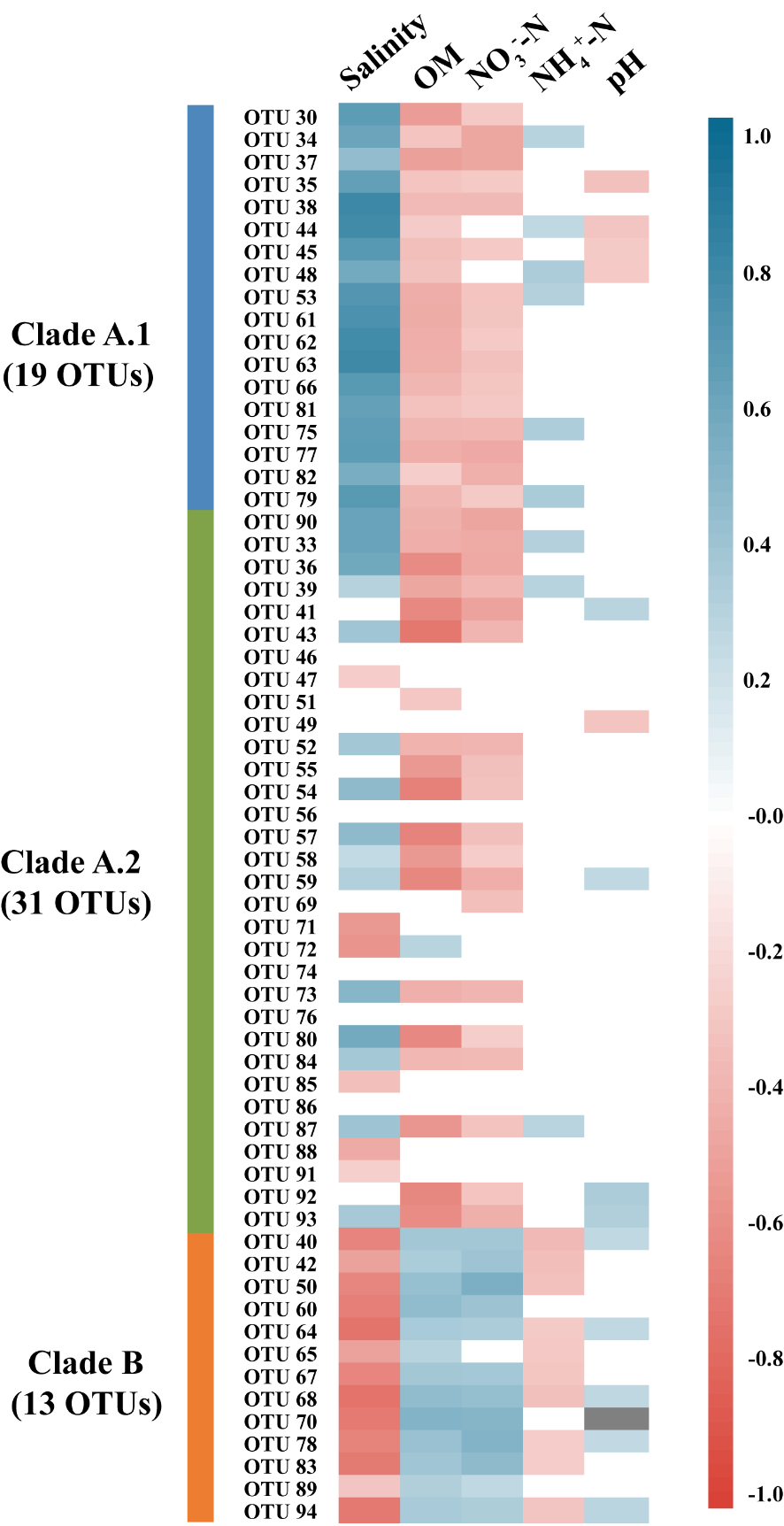


**Supplementary Figure S4 Spearman correlations of lower abundance OTUs (0.1% < abundance < 1%) of comammox *Nitrospira* with soil properties in all samples, displayed as heatmaps.** Scale bars indicate correlation coefficients. Only significant correlations (p < 0.05) are shown.


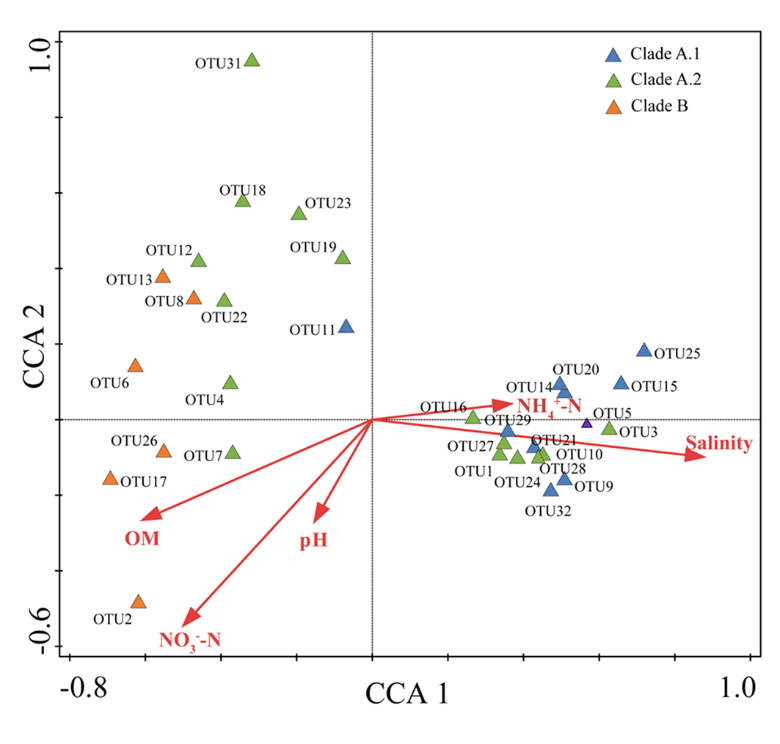


**Supplementary** **Figure S5 Canonical correspondence analysis (CCA) of comammox *Nitrospira amoA* communities and environmental variables.** Two axes of the CCA explained 28.48% of the total variance and 80.11% of the cumulative variance of the genotype-environment relationship.


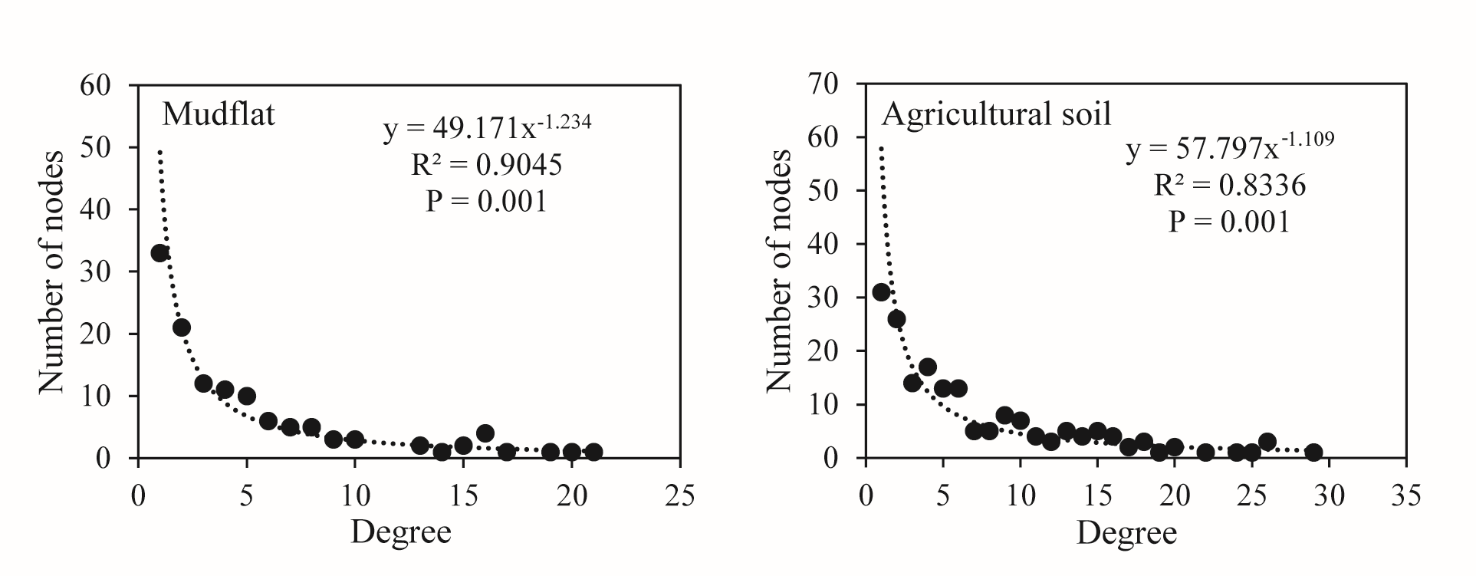


**Supplementary Figure S6 Network topological features of degree distribution patterns in mudflat and agricultural soil.**
